# Error Fields: Personalized robotic movement training that augments one’s more likely mistakes

**DOI:** 10.21203/rs.3.rs-3165013/v1

**Published:** 2023-07-14

**Authors:** Naveed Reza Aghamohammadi, Moria Fisher Bittmann, Verena Klamroth-Marganska, Robert Riener, Felix C. Huang, James L. Patton

**Affiliations:** 1 Robotics Laboratory, Center for Neural Plasticity, Shirley Ryan AbilityLab, Chicago, IL, USA; 2 Department of Biomedical Engineering, University of Illinois at Chicago, Chicago, IL, USA; 3 Department of Health Sciences and Technology, Swiss Federal Institute of Technology, Zurich, Switzerland; 4 Spinal Cord Injury Center, Balgrist University Hospital, University of Zurich, Zurich, Switzerland; 5 Department of Mechanical Engineering, Tufts University, Medford, MA, USA

## Abstract

Control of movement is learned and uses error feedback during practice to predict actions for the next movement. We have shown that augmenting error can enhance learning, but while such findings are encouraging the methods need to be refined to accommodate a person’s individual reactions to error. The current study evaluates *error fields (EF)* method, where the interactive robot tempers its augmentation when the error is less likely. 22 healthy participants were asked to learn moving with a visual transformation, and we enhanced the training with error fields. We found that training with error fields led to greatest reduction in error. EF training reduced error 264% more than controls who practiced without error fields, but subjects learned more slowly than our previous error magnification technique. We also found a relationship between the amount of learning and how much variability was induced by the error augmentation treatments, most likely leading to better exploration and discovery of the causes of error. These robotic training enhancements should be further explored in combination to optimally leverage error statistics to teach people how to move better.

## Introduction

Making mistakes is inherently human, and learning from them may require more salient experiences to enable the best improvements in performance. Prior research has successfully shown enhanced motor learning using error augmentation (EA) as a training technique. This technique artificially exaggerates the visual or haptic feedback of the movement errors. Because error feedback is of particular importance to motor learning, enhanced error drives enhanced changes to the feedforward plan^1,2^. A variety of tasks have been shown to mildly benefit from using error augmentation techniques, including upper^3–6^ and lower extremity activities^7–10^. Like other forms of interactive virtual environments, the novel experience of error augmentation could serve to increase overall engagement to promote improvements in performance. Attention, error, and reward are essential for forming and reinforcing memories of motor experience. However, sensorimotor manipulations that specifically make errors more noticeable improve the rate and amount of motor learning^11^. In fact, error augmentation promotes learning gains that are not present in assistive approaches^12^. On the other hand, studies suggested that augmenting with gains outside of an appropriate range is detrimental to learning^13,14^. Evidently, excessively large or spurious errors diminish the adaptive response^15^.

The inherent challenge of designing intensive training is to promote learning that is relevant outside of training^16–19^. The motor system combines multiple sources of information in a statistically optimal fashion^20,21^, so learning from the strangeness of augmented feedback must be somehow reconciled with the learning of lifelong experiences. Error augmentation, however, naturally provides a smooth transition since sufficient training should reduce errors enough so that nothing remains to be augmented. Even so, recent studies show that motor learning and successful skill transfer are influenced by the statistics of past experiences of error feedback^22,23^. Hence, unexpectedly large signals during a training session could disrupt learning. Yet these findings suggest an exciting possibility: could the statistics of error provide engineering guidelines for how to focus training? Such streamlined augmentation might reinforce existing statistical processing^22^, while at the same time presenting only minimal distortion to sensorimotor experiences.

Rather than determining a single level of training intensity that fits most learners^13^, a more sensible approach might be to incorporate individual needs into the design of training. Studies have shown success in flexible strategies for robot-assisted rehabilitation by gradually increasing load^24^, adapting forces as needed^25^, or simply allowing participants to choose therapy models^26^. However, such methods that use a *Challenge Point* Framework that suggests training difficulty should be enhanced to match the learners initial success level^27,28^.

Similarly, in therapy, customization is especially vital to address wide-ranging differences in impairments. Our research group already has shown that the initial patterns of abnormal coordination can be characterized and then reshaped with custom forces^29–31^. Similarly, we found that the errors seen during goal-directed reaching can be described in terms of simple probability distributions^32–35^. Characterizing the statistics of an individual’s reaching movement effectively allows not only a scientific assessment of specific repeatable deficits but it should also allows us to make use of such error likelihood and custom-design effective training.

Here we evaluated this new form of error augmentation that is customized according to each individuals specific error tendencies. Our approach begins with a statistical profile of error trajectories for a given participant. We then formulate an algorithm that augments error only in regions of high probability of error. We conducted an experiment in which participants learned to make straight-line reaches in a visually distorted space. The motion of the on-screen cursor was driven by joint angles of the shoulder and elbow similar to that of Flanagan et al.^36^. This task required participants to improve both spatial and extent (timing) errors during reaching. Our research question hypothesized that this new error field treatment (EF) would provide faster learning and improved skill transfer to non-augmented conditions. Study results were presented in preliminary form^37^, and here we include an examination of learned behaviors customized for each participant.

## Methods

### Participants

**Figure 1.**
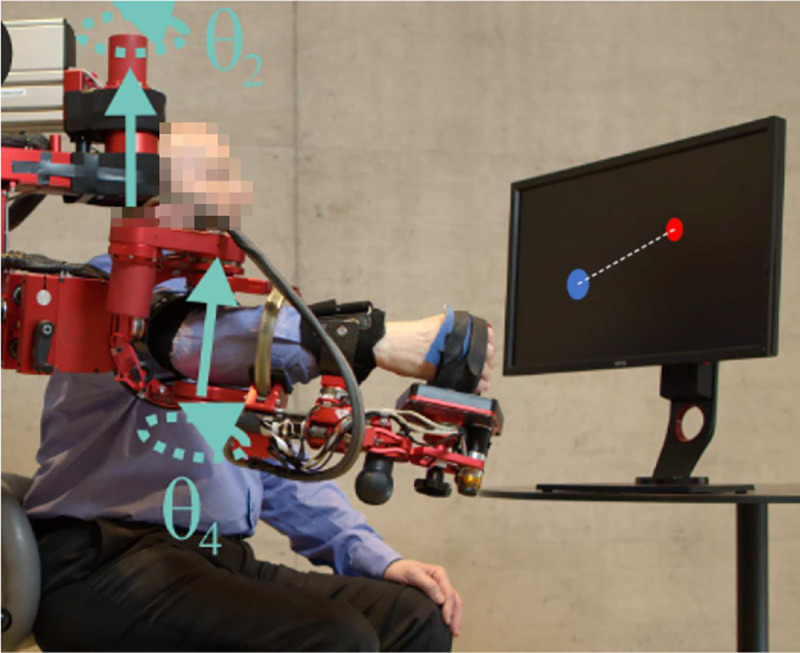
Participants performed a planar reaching task using the ARMin exoskeleton device restricted to shoulder rotation and elbow extension. ARMin device was locked in a planar configuration with targets displaced on a vertical computer screen, here shown with line connecting start of movement to target(blue circle)

This experiment employed a seven degrees-of-freedom robotic arm exoskeleton device, the ARMin III (Figure 1), located at the Sensory-Motor Systems Lab at ETH Zurich (Zurich, Switzerland)^38^. Twenty-two right-handed healthy participants (10 female) with no prior neurological impairments. Participants were randomly assigned to one of three experimental groups with 8 participants in the group that received EF training and 7 in the other two groups. Subject number 4 was removed from analysis after discovering that speed instructions were ignored across the experiment.

Experimental protocols were approved by the Ethics Board of Canton Zurich. All methods followed guidelines and regulations set by the Ethics Board of the Canton Zurich and were in accordance to the ethical standards of the Declaration of Helsinki. Informed Consent was obtained from all subjects in accordance with the Ethics Board of Canton Zurich.

### Experimental Protocol

We investigated motor learning of a novel visuomotor transformation as the test-bed for evaluating the training benefits of augmenting error feedback. Participants were asked to perform goal-directed reaching movements using the robotic exoskeleton with their right arm. In normal reaching conditions and after healthy participants have become familiarized with the robotic device, deviations from the straight line were negligible. We first confirmed this by checking their late-baseline trajectories and the deviations were not significantly different from a straight line.

To facilitate planar reaching, a proportional-derivative controller was used to orient the robot such that only shoulder rotation $\theta_{2}$ and elbow flexion/extension $\theta_{4}$ were possible (Figure 1). Known friction, gravitational, and viscous effects of the robot were compensated by controller to make the robot haptically transparent to the user.

To introduce a challenging motor task, we selected a visuomotor transformation similar to^36^. In this environment, rather than x-y hand position, we displayed a plot of shoulder vs. elbow angle on a screen 1.5 meters anterior to subjects’ heads (Figure 1). This visual transformation required participants to learn a visuomotor remapping to move the cursor on this angle-angle plot.

For each trial, the task required participants to move along a straight-line path between two targets located 15 centimeters apart on the screen (See Figure 1). Participants were instructed that they could begin the reach as soon as the target appeared on the screen. Participants were allowed to rest in the target before initiating the next reach. After completion of a reach, feedback on movement time was given. We assumed an ideal reach duration of $t_{d}=1.5$ by averaging different subjects from pilot studies in normal conditions (i.e., no visual distortion) in this lab. Subjects were given duration feedback at the end of each movement, which created equitable conditions for the required speed across all trials. Specifically, an ideal reach duration of 1.5 seconds resulted in the target turning green when the trial was proper timing, red if too slow, and yellow if too fast.

**Figure 2.**
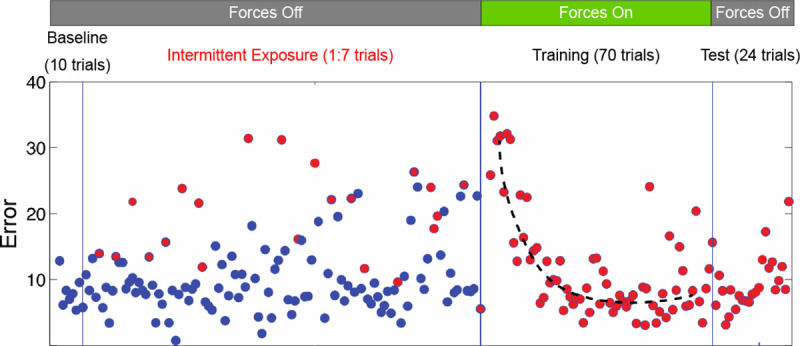
Experimental protocol and representative errors across phases for a single participant from the EF Group. Blue circles represent baseline reaching and red circles represent reaching in joint-angle transformation. *“Forces Off”* means that no force was applied to the hand, and *“Forces On”* means that treatment forces were applied. During training, the black dashed line represents the trials used to measure the time constant of error decay (equation (3)). Definitions of errors are explained in methods section.

During Baseline (See Figure 2), participants first familiarized themselves with the device with normal intuitive endpoint visual feedback (in Cartesian coordinates). During Intermittent Exposure, we presented participants with the angle-angle visuomotor transformation, randomly presented one in five trials, in a variety of movement directions. This randomized schedule revealed each participants initial (poor) performance prior to the training phase. All participants experienced a short pause after Baseline prior to commencing training with the angle-angle visuomotor transformation for seventy trials.

### Error measurement

**Figure 3.**
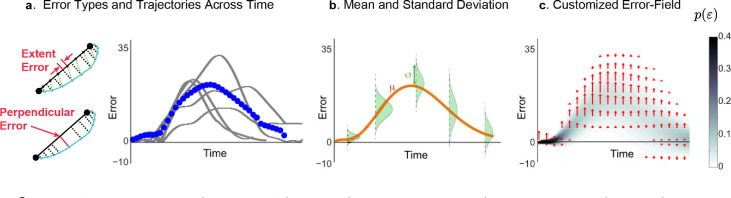
Example application of the Error field (EF) technique. (a) Prior to the main training phase of the experiment, participants were presented with intermittent trials that featured the novel control that mapped joint angle to screen space. The average errors (blue dots) and standard deviation were computed across the trajectory for each participant. (b) The mean μ and standard deviation σ were fit with 7^*th*^ order polynomials, which enabled a normal distribution to be formulated at each sample in time. (c) The error field was then designed to apply torque (indicated by red arrows) according to that magnitude and probability of errors in separate functions for extent and perpendicular errors.

We assumed that the ideal movement was a straight line from the starting point $x_{o}$ to the target $x_{T}$, following the minimum jerk velocity profile^39^,

(1) $$x_{MJ}\left(t\right)=x_{o}+\left(x_{T}-x_{o}\right)\left(10{\left(\frac{t}{t_{d}}\right)}^{3}-15{\left(\frac{t}{t_{d}}\right)}^{4}+6{\left(\frac{t}{t_{d}}\right)}^{5}\right)$$

where $x_{MJ}$ was position, optimized for motion smoothness arriving at the target at the terminal time $t_{d}$ of 1.5 s. Error vectors were resolved to *extent* (in the target direction) and *perpendicular* components (Figure 3a). Our primary outcome was the reduction of error magnitude, but we also investigated secondary measures to further understand more aspects of learning.

### Error Field Treatment

The novel visual transformation presented during Intermittent Exposure Phase allowed us to formulate a statistical distribution for each participant, estimating the 2-dimensional probability of error. A normal Gaussian model at each time instant resulted in a time-based function p(ε) across the error space^32^. The following steps describe the technique:

1. Error vectors $\varepsilon_{i}$ were computed along each trajectory, then grouped for each of the 3 movement directions d, where the subscript i indicates the error vector components (*extent* and *perpendicular*).
2. Intermittent Exposure trials were used to evaluate error probability p(ε) by calculating Mean and Standard Deviation.
3. We fit Seventh order polynomials error means $\mu_{d,t,i}$ and standard deviation $\sigma_{d,t,i}$ trajectories across time for the first 1.5 seconds of movement. This resulted in a typical Gaussian expression for the probability of error p(ε) for each time step t, movement direction d, and error vector component i (extent, perpendicular),
(2) $$p(\varepsilon )=\frac{e^{\frac{-{\left(\varepsilon -\mu_{d,t,i}\right)}^{2}}{2\sigma_{d,t,i}^{2}}}}{\sigma_{d,t,i}\sqrt{2\pi }}$$
4. Multiplying this instantaneous error likelihood p(ε) by the error ε forms the *error field* that determines the robotic torque generated in real-time (Figure 3c),
(3) τ=λp(ε)ε
  where τ and ε are vectors and the scale factor λ was determined so that 80% of estimated peak torques would reach 15 Newton-meters. This was based on torques tolerated by subjects in our prior experiments on neurotypical individuals^11,17,40^. λ was constant throughout the entire training phase.
  In contrast, the EA group only received torques proportional to the error vector:
(4) τ(t)=λε

Note torque τ and error ε were treated as vectors with components in the direction to the target (“extent”) and its perpendicular, while probability p(ε) is a scalar. Torques were presented only during the first 1.5 seconds of movement so that participants were able to reach the target and complete the movement. Torques were also limited to 15 Nm for safety limits.

### Analysis

**Figure 5.**
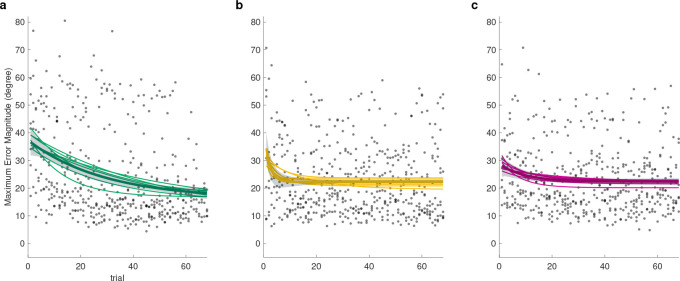
Learning regression curves for the (a) EF, (b) EA, and (c) Control groups. Shaded areas indicate 3 × *SD* of the fit (thick solid line) that incorporated all data, other thinner solid lines represent the fits of one-subject-leave-out cross-validation. The parameters of these fits are represented by the dots in Figure 6.

In order to assess performance, i.e., the speed and amount of learning in groups, we used non-negative least squares regression to identify a simple three-parameter exponential error decay across training similar to^5,16^. The model is $S+Re^{-t/\tau }$, where S is the steady-state error (if participants were given infinite trials during training), t is trial number, R is error reduction, and τ is the time constant of the fit (representing the speed of learning). Cross-validation ensured robustness; one fit with data from all the subjects in that group and then seven fits with removing one subject’s data; each fit had 50 repeats with 20% leave-out for testing; The best fit was selected by evaluating Normalized Root Mean Square Error cost function on the test data; see Figure 5. In order to calculate each subject’s error reduction across training, we used a non-parametric measure of calculating the mean of the first five error values at the beginning of training minus the mean of the last five error values in training; we called this measure “Change in Error”; see Figure 7. Data were checked for normality using Shapiro-Wilk^41^ test. In the second stage, MATLAB software examined significance levels using Kruskal-Wallis^42^ test and followed by pairwise post hoc tests with Dunn-Sidak^43^ corrections. With these, we hypothesized a-priori that EF training would result in the lowest error (α=0.05).

## Results

### Models of error reduction

**Figure 6.**
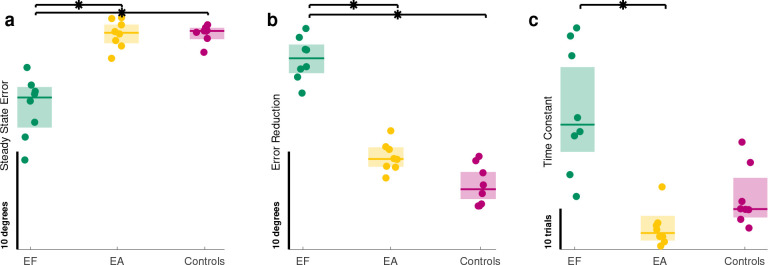
The simple parameters of a model describing benchmarks for learning in different groups. Each dot represents a parameter from the equation $S+Re^{-t/\tau }$ fitted to all or a subset of subjects error during training, the shaded box shows 95% confidence intervals, the horizontal line shows median of that group: (a) Steady State Error (S) in which EF group outperformed others, (b) Error Reduction (R) in which EF group outperformed (c) Time Constant (τ) for which EA learned fastest.

We found that training with error fields (EF) was indeed the superior method for error reduction (Figures 5 and 6). The EF training group resulted in the lowest steady-state error(Figure 6a) as evidenced by main effect of group (Kruskal-Wallis df=2;F=15.38;p=0.00046). Post hoc contrast revealed that steady-state error was 73% lower than Controls (p=0.00159) and 72% lower than EA (p=0.0027). The EA group averaged only (2% lower) than controls (p=0.99). Please refer to supplementary document for detailed tables of results as well as individual subject performances.

These steady state results corresponded with error reduction (Figure 6b). The EF training resulted in highest amount of error reduction as supported by main effect of group(Kruskal-Wallis df=2,F=18.005,p=0.00012). Post hoc comparisons revealed that error reduction of EF group was 264% more than controls s $\left(p=7.75\times 10^{-5}\right)$ and 80% more than EA by (p=0.0293). EA also averaged 102% more than controls, but not significantly (p=0.28).

We also analyzed how long it took to learn, measured by the time constant τ (Figure 6c). Significance was observed in the main effect of group (Kruskal-Wallis: df=2,F=16.305,p=0.00029). Interestingly, the EF group averaged 65% longer compared to Controls (p=0.075). EF was also 253% slower than EA $\left(p=1.6\times 10^{-5}\right)$. Consistent with previous research^13^, EA was 53% faster than Controls, but not significantly (p=0.855).

### Error Variability

**Figure 7.**
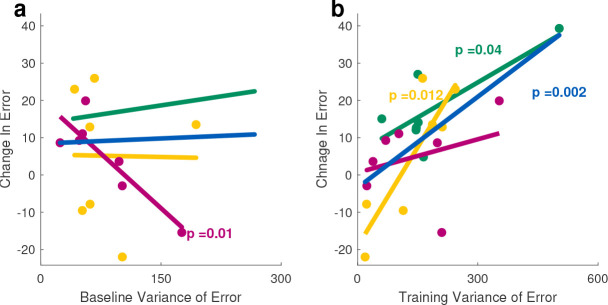
Variability of error amplitude can partially explain the individual subject’s change in error during the training. Solid colored lines represent a linear correlation model in a group(Green: EF, Yellow: EA, Purple: Controls and Blue representing all subjects in all groups),(a) Change in error is not correlated with baseline variability, (b) Change in error is correlated with variability during training, but not for controls.

While EF was not designed to change variability, it was plausible that a novel environment could expand trial-to-trial variations, and such variations are suggested to be an important pathway in motor adaptation^44,45^. Alternatively, EF may reduce variance because it is customized to only enhance the most typical errors. We first inspected how each individual’s natural variation (during baseline) was related to the their error reduction during training. We observed no evidence of a correlation between baseline variability and change in error (ANOVA for each linear model fit, significant p-values are shown in Figure 7a). However, for the EF and EA groups, subject error reduction was significantly correlated with their training variability (Figure 7b). This correlation was not observed for the control subjects, and helped to contrast the effect of the haptic error augmentation treatments (EF and EA) compared to visual distortion seen alone.

Finally, we speculated that error field training may allow specific predictions. For EF, error should shift away from its initial tendencies to avoid the EF forces, but may not necessarily reduce errors to zero mean. We measured this shift of error ensemble distribution Δε(t), from early to late training, and normalized it with respect to the standard deviation of early training distributions. We found that for EF group alone we could readily predict the shift in mean error to be reliably 1.3 standard deviations from the original error.

## Discussion

This study evaluated a novel method of personalized robot-assisted training for motor skills, based on error probability. We found that such eror field (EF) training led to greater error reduction − 264% more. However, participants of our previous technique, error augmentation (EA), learned faster. The EF technique focused on *repeatable* errors, where spurious or infrequent errors became de-emphasized. We expected that this type of training would allow the motor system to be more sensitive to errors they repeatedly make. EF did not outperform our previous EA) techniques to enhance motor learning^4,5,7,13,46,47^. In contrast to these other studies, the current approach modulates error augmentation based on the likelihood of error, in the space of movement errors. This design was motivated by the idea that the augmented feedback should bear some similarity to an individuals previous motor learning experiences.

The *error space* is important to learning, and the key contribution of EF was that error the augmentation is small only if that error has not been experienced before. This personalized form of error augmentation demonstrates how to incorporate statistics into robotic training. Studies suggest probabilistic processing is an inherent part of the motor system^21,48^. In such Bayesian learning models, movement errors that are similar to expectations would drive adaptation, while spurious errors would be given little weight. We believe that our intervention supports this learning process by emphasizing useful information (in this case, the most consistent reaching errors). In response to a varying environment, humans can combine an appropriate feedforward and impedance control^49^. Herzfeld and colleagues^23^ showed that prior experience of error, in the error space, governs the amount of learning. Furthermore, researchers have shown that providing feedback related to the frequency of previous errors can improve the rate of adaptation^50^. In our current paradigm, we are ensuring that the more likely errors are amplified during training. These changes align with recent findings from Takiyama and colleagues showed that people learn most when they have made that error before^51^.

Inspecting variability (Figure 7) also displayed its relation with error reduction. We speculate that this is because the error likelihood profile was unique to each subject, varied, and.^44,45^ Many studies of goal-directed movements focus on models of adaptation based on trial-to-trial error. Our findings suggest that a more comprehensive approach would be to consider how probabilities of error throughout the trajectory changed across learning. Beyond variation between individuals, it was clear that *within* an individual the error likelihood varies along the trajectories and can also be different for each movement direction (See Fig. 3). It remains to be seen whether these directional differences are neural or biomechanical (or both).

While the magnitude of learning showed its advantages, time to learn (speed of learning) did not offer advantages for EF. EA treatments were *fastest* (Figure 6c). Movement-to-movement variability (of error) may be a reason. We did also explore several measures, such as fitting a simple exponential decay process^5,16^, estimating settling time, and inspecting discrete-time auto-regressive models^52^. These methods yielded similarly ambiguous results that failed to show the superiority for learning speed seen in previous studies^13^. Late-training variability was high, causing models to over fit, identify unrealistically slow time constants, or produce unstable auto-regressive models. This mainly happened when subjects had little to learn, having good initial performance and subsequent errors were variability. Further testing may reveal tasks that can produce “cleaner” data to help distinguish these different error augmentation treatments.

We used a simple visuomotor distortion that required a particular type of learning – a remapping of sensory (vision) to motor output. For this, participants could not simply increase their impedance via muscle co-contraction to resist the distortion. This is in contrast to force field adaptations, where movement accuracy can be helped by co-contractions^53,54^. Here the distortion required learning altered motions rather than forces, so impedance adaptation can be ruled out.

Another possible concern is that lower overall torques in the EF condition might present advantages in terms of lower workload. However, we scaled the magnitude of applied torques for each group according to the errors observed during intermittent exposure. If anything, loading should be the least for the EF condition because their forces were tempered by error likelihood. Nevertheless, no significant differences in force levels were detected between groups.

The differences we found highlight how combined augmentation types may lead to even better learning. Previous studies showed learning improvements^3,5,16,27^. Here we observed an advantage of the EF treatment over the previous EA technique for extent component of error (Fig. 6a). While error reduction was more, we failed to detect a significant advantage in *reduction rate* from the EF treatment; if anything, the opposite was true. It remains to be sen whether some combination of forms may be used interchangeably to target a specific goals for the learner.

The fact that error reductions differed by component^55^ also suggests a form of performance optimization, where some variables were prioritized at the cost of others^56^. The motor system might prefer straight-line movements given strong visual feedback represented as a Cartesian space^57^. In this task, extent errors are essentially timing errors that do less to change task outcome. However, we believe that the added benefit of our treatment focused subjects’ attention on extent, allowing participants in the EF Group to selectively decrease a distinct component of error that would not otherwise have been possible. In other words, the EF approach might be used to bias learning tendencies within the redundant space of possibilities, as suggested other theories such as the Uncontrolled Manifold Hypothesis (UCM)^58^ and body machine interfaces^59^.

While the error field treatment resulted in the most learning in this study, residual errors still remained at the end of training. This pattern of incomplete learning was likely due to the fact that the customization was based only on the initial characterization phase and did not reflect changes in error patterns during training. To truly capture the challenge point^28^ required for optimal learning and ultimately drive error to zero, we would need to re-characterize error as training progresses. Furthermore, one disadvantage of our current implementation of error fields is that it assumed symmetry in error about the mean trajectory. Future implementation of this technique might feature non-normal distributions, or additional Gaussian components for characterizing error as well as continually updating error fields as the training progresses. Such strategies would ensure that smaller distributions of error that occur later in training will also be further driven towards zero error. Re-characterization techniques have been successful both with adaptive resistance^60^ and assistance training^26^. Another important consideration is that while this study chose to parameters error distributions as a function of time, many other choices could easily be implemented. Our current design was inspired by our previous study that showed better fit across time compared to across state^15^.

This paper represents a critical step in training design methodology. The approach we used can easily be adapted for specialized tasks whenever one desires to accommodate wide differences in movement errors across subjects, even when neurophysiological or biomechanical sources of error are unknown. Note that the error fields considered in this study accommodated differences across the trajectory as well as movement direction. This design provided a detailed level of customization (or personalization), more than simply adapting to each subject. We believe that this strategy of focusing the intervention on error probabilities is applicable to any training situation where error can be measured and characterized. Beyond enhancing training, our findings support the idea that the nervous system relies on statistical error information for learning which could extend to additional tasks that have a defined goal or trajectory. Understanding such neural mechanisms of learning could then provide both inspiration and a mathematical algorithm for better training interventions. We hope this work might inspire others in a variety of fields, including neurorehabilitation, robotics, sports training, music performance, education, piloting, or surgical training.

## Figures and Tables

**Figure 4.**
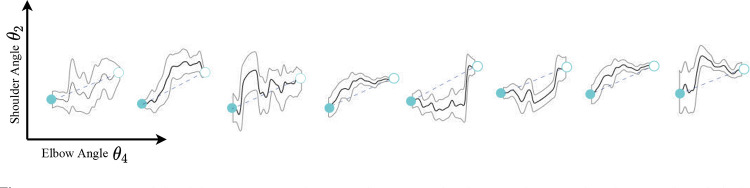
Participants exhibited different error tendencies with respect to the ideal straight-line path to the target (blue dashed line). Dark lines indicate the mean trajectory and gray lines indicate 95% confidence intervals for all participants in the Error Field Group.

## Data Availability

The data sets analysed during the current study are available in the Dryad repository^61^, https://doi.org/10.5061/dryad.prr4xgxnv.
